# The utility of the rodent synergist ablation model in identifying molecular and cellular mechanisms of skeletal muscle hypertrophy

**DOI:** 10.1152/ajpcell.00362.2024

**Published:** 2024-07-29

**Authors:** Benjamin I. Burke, Ahmed Ismaeel, John J. McCarthy

**Affiliations:** ^1^Department of Physiology, College of Medicine, University of Kentucky, Lexington, Kentucky, United States; ^2^Center for Muscle Biology, University of Kentucky, Lexington, Kentucky, United States

**Keywords:** microRNAs, mTOR signaling, protein synthesis, ribosome biogenesis, satellite cell fusion

## Abstract

Skeletal muscle exhibits remarkable plasticity to adapt to stimuli such as mechanical loading. The mechanisms that regulate skeletal muscle hypertrophy due to mechanical overload have been thoroughly studied. Remarkably, our understanding of many of the molecular and cellular mechanisms that regulate hypertrophic growth were first identified using the rodent synergist ablation (SA) model and subsequently corroborated in human resistance exercise training studies. To demonstrate the utility of the SA model, we briefly summarize the hypertrophic mechanisms identified using the model and the following translation of these mechanism to human skeletal muscle hypertrophy induced by resistance exercise training.

## Graphical abstract

**Figure:**
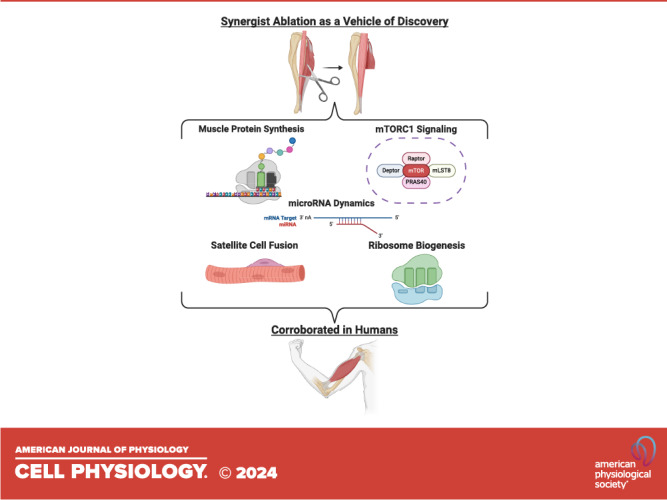

## INTRODUCTION

**Figure 1.**
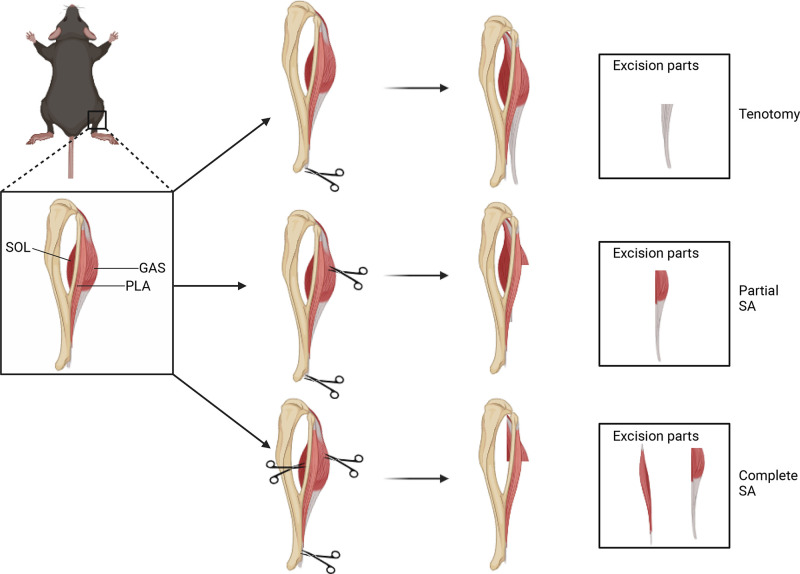
Different models of mechanical overload. Mouse hindlimb anatomy shown, highlighting gastrocnemius complex consisting of gastrocnemius (GAS), plantaris (PLA), and soleus (SOL) muscles. Excision parts for tenotomy—GAS tendon; partial synergist ablation (SA)—2/3 GAS; complete SA—soleus, 2/3 of GAS. Created with Biorender.com.

In 1967, Alfred Goldberg first described a model of rodent hypertrophy where the gastrocnemius tendon was transected (tenotomy) to induce compensatory hypertrophy of the synergistic muscles (i.e., soleus and plantaris) by mechanical overload (MOV) (1, 2). Since the initial model, different surgical approaches have been used to induce mechanical loading of the plantaris and soleus muscles (Fig. 1). Nearly 60 years later, this model, now aptly coined synergist ablation (SA), has been widely used in the exercise physiology field to better understand the molecular and cellular mechanisms of skeletal muscle hypertrophy (3, 4). The SA model induces a robust increase in muscle size due to chronic MOV (5) and has revealed the importance, as described below, of protein synthesis, mTORC1 signaling, and ribosome biogenesis in muscle hypertrophy (6). Importantly, the SA model has provided the fundamental knowledge that mechanical loading is the primary factor through which resistance exercise training promotes muscle hypertrophy in humans.

Despite the undeniable success of the SA model in the identification of molecular and cellular mechanisms involved in the regulation of hypertrophy, critics remain. The most common critique of the SA model is its failure to accurately mimic resistance exercise in humans (3, 5). Indeed, the SA model places a chronic, “round-the-clock” MOV on the synergist muscles. This chronic MOV induces a “supraphysiological” muscle growth (3, 5, 7). Although the approximately twofold increase in muscle mass following 14 days of MOV is no doubt impressive, it is eclipsed by the almost sevenfold increase in muscle size during the 21 days of postnatal development in the mouse (8). These concerns regarding the pertinence of the SA model to human skeletal muscle growth in response to resistance exercise training have led to the development of alternative models of rodent hypertrophy with the goal of more closely mimicking resistance exercise training (3, 9–14). Although these more “translatable” models have been shown to be effective in inducing hypertrophy, they remained limited for precisely the reasons the SA model has been so successful at discovering the mechanisms of muscle hypertrophy. First, the robust nature of chronic MOV compresses the time scale upon which molecular and cellular events occur, thus enhancing the practicality of the model. Second, the response to MOV is highly reproducible, though this can be influenced by the activity level of the mouse. Finally, chronic MOV induces such robust responses that the detection of relevant molecular and cellular events above the biological “noise” is enhanced. Collectively, these characteristics of the SA model allow for precise mechanistic investigation such that a detailed time course response to SA has been characterized. For example, it is now well-established that in response to SA in mice, preinitiation complex formation for ribosome biogenesis occurs at *day 3* of MOV, whereas the majority of satellite cell fusion occurs at *day 7* of MOV (15, 16).

The purpose of this brief review is to provide evidence for the utility of the rodent SA model for identifying mechanisms of skeletal muscle hypertrophy. Below, we summarize the fundamental mechanisms identified using the SA model and subsequently corroborated in human studies. We also present promising new discoveries that occur during SA-induced hypertrophy that remain to be validated with resistance exercise training studies in humans.

## MECHANISMS DISCOVERED USING SYNERGIST ABLATION MODEL

### Muscle Protein Synthesis

On the heels of an emerging field of protein synthesis (17), Goldberg’s newly developed SA model was used to demonstrate for the first time in vivo that skeletal muscle hypertrophy is associated with a proportionate increase in muscle protein synthesis (1, 18). It was soon accepted that skeletal muscle growth is characterized by positive nitrogen balance (protein synthesis exceeding protein degradation) (19). Before the turn of the century, it was confirmed in humans that muscle protein synthesis increased in response to resistance exercise (20). Although it was many years between the papers establishing the importance of muscle protein synthesis and the identification of the signaling pathways regulating protein synthesis (see below), this pioneering discovery laid the foundation for the field to build an understanding of skeletal muscle hypertrophy.

### Mammalian Target of Rapamycin Signaling

The protein kinase mammalian target of rapamycin (mTOR) was first described in 1994 by Brown et al. (21) who demonstrated its mediatory effects on cell cycle progression. Before long, mTOR was implicated in insulin signaling (22), adipogenesis (23), cancer (24), and more (25). Following up on their in vitro work establishing the importance of the Akt/mTOR signaling pathway in regulating skeletal muscle hypertrophy (26), Bodine et al. (27) published a seminal paper confirming and expanding these findings in vivo using SA as a model of hypertrophy. Literature quickly amassed, elucidating the mechanisms by which mTOR signaling regulates protein synthesis to drive muscle hypertrophy and establishing the indispensable role of mTOR in skeletal muscle hypertrophy (28–30). Not surprisingly, many of the early studies used the SA model (27, 30–32). Shortly thereafter, these findings were validated in human resistance exercise training studies, cementing mTOR signaling in the pantheon of fundamental mechanisms of skeletal muscle growth (33, 34).

### Ribosome Biogenesis

With the importance of protein synthesis in skeletal muscle hypertrophy established, efforts began to interrogate the translational machinery in relation to muscle hypertrophy. Early work demonstrated that ribosomal RNA was elevated with MOV (35) and, conversely, ribosomal RNA and content were lower with disuse (36). Although it was clear that translational capacity (i.e., ribosome content) was increased during muscle growth, the mechanism regulating ribosome biogenesis was unknown at the time. Using the SA model and in vitro models of muscle hypertrophy, it was discovered that the activation of mTORC1 signaling and subsequent ribosomal p70 S6 kinase phosphorylation is a driving factor in ribosome biogenesis (29, 37). These findings were later corroborated in humans (38), demonstrating yet again the power of the SA model in elucidating fundamental processes in skeletal muscle hypertrophy.

### Satellite Cell Fusion

In the rapidly expanding field of satellite cell dynamics and muscle regeneration, researchers noted an increase of satellite cells with muscle hypertrophy induced by SA (39, 40). Shortly thereafter, it was discovered that, in response to a hypertrophic stimulus, satellite cells become activated, replicate, and then fuse to myofibers, leading to an increase in myonuclear abundance (41). Confirming these findings, it was soon discovered that satellite cells proliferate in response to resistance exercise in humans as well, which is associated with an increase in myonuclear abundance, presumably as a result of satellite cell fusion (42, 43). Due to the robust satellite cell response to SA, this model has proven to be a useful technique for studying satellite cell dynamics (44, 45).

### Muscle-Specific microRNAs

Shortly after the discovery of microRNAs (miRs) as posttranscriptional regulators of gene expression, several tissue-enriched microRNAs were identified, including the muscle-specific miR-1, miR-133a, and miR-206, later designated as muscle-specific microRNAs (myomiRs) (46, 47). SA was first used to show that the expression of the canonical myomiRs was changed in response to a hypertrophic stimulus; specifically, miR-1 and miR-133a expressions were significantly lower after 7 days of MOV (48). Later, an acute bout of resistance exercise was shown to downregulate miR-1 expression in humans (49, 50). Interestingly, miR-1 and miR-133a were also found to be less abundant in powerlifters (51). As the role of miRNAs in skeletal muscle hypertrophy continues to be of interest to the field (52) and the function of myomiRs is still being elucidated, the SA model is expected to remain a powerful tool for uncovering the mysteries of the myomiR network (50, 53, 54).

## PROMISING NEW FRONTIERS ESTABLISHED USING SYNERGIST ABLATION

### Intertissue Cross Talk

Extracellular vesicles (EVs) have emerged as important mediators of intertissue cross talk. EVs have also been identified as exercise-induced mediators of intertissue signaling and exercise adaptation (55). Skeletal muscle has been shown to be an important source of myomiR-containing EVs following exercise (56). MOV induced by SA was shown to result in EV-mediated miR-1 trafficking from skeletal muscle to adipose tissue, which enhanced lipolysis via altered ß-adrenergic signaling (50). Whether or not a similar mechanism is operative in humans remains to be determined.

### Epigenetics

SA has been used to study the skeletal muscle DNA methylation landscape during muscle remodeling (57–59). These studies have identified hypomethylated promoter regions of several genes involved in growth, including mTOR signaling and autophagy regulation. Moreover, the promoter region of Myc was shown to be hypomethylated following SA-induced mechanical overload (57–59). Further research is needed to establish the epigenetic regulation of skeletal muscle hypertrophy in humans.

### Metabolic Reprogramming

Beyond elevated protein synthesis, hypertrophying cells also rely on other anabolic reactions, including nucleotide synthesis, lipid synthesis, and synthesis of substrates for epigenetic modification (i.e., acetyl and methyl groups). Recent research suggests that muscle hypertrophy may be characterized by metabolic reprogramming toward aerobic glycolysis (i.e., the Warburg effect) to channel glycolytic intermediates toward biosynthetic precursor production (60). Interestingly, SA in mice was found to result in downregulation of oxidative metabolism gene expression (57) and reduced mitochondrial respiration (61), whereas glycolytic flux is increased (62). Moreover, SA induces upregulation of serine-synthesizing enzymes, including phosphoglycerate dehydrogenase (Phgdh) (63). SA also leads to the upregulation of glucose-6 phosphate dehydrogenase (G6pd) and activation of the pentose phosphate pathway (53, 62). Whether human muscle hypertrophy in response to resistance exercise training may also be associated with redirected glycolytic flux toward biosynthetic pathways remains to be elucidated.

### Satellite Cell Dynamics

Novel roles of satellite cells in load-mediated growth are beginning to emerge using the SA model (16). Specifically, intercellular delivery of EVs to fibrogenic cells, endothelial cells, and myofibers has been shown to affect hypertrophic adaptation (16). Thus, in addition to fusion and myonuclear donation, satellite cells likely coordinate exercise adaptation via secreted factors. SA has also been used as a model to study other aspects and mechanisms of satellite cell dynamics. Fukada et al. (64) have identified activation and proliferation factors that regulate satellite cell proliferation and differentiation in response to MOV using the SA model. Furthermore, our laboratory identified a novel mechanism of division-independent differentiation of satellite cells in response to SA, whereby a significant proportion of satellite cell-derived myonuclei differentiated and fused into muscle cells without undergoing DNA replication (65). Although these pathways have yet to be established in hypertrophying human muscle, SA provides an alternative model to study satellite cell behavior in adulthood that is different from development and regeneration.

## CONCLUSIONS

**Figure 2.**
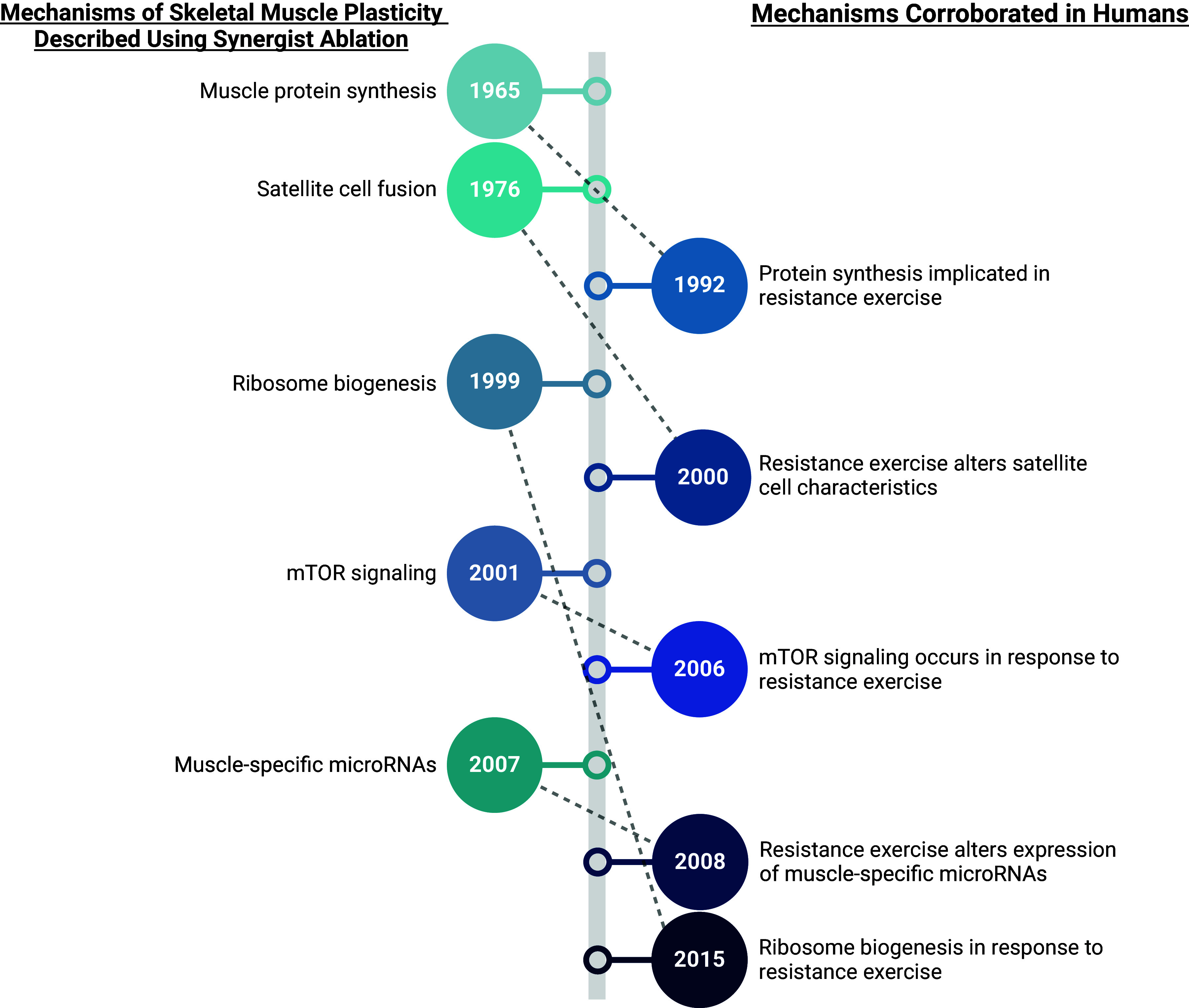
Synergist ablation discovery timeline. Timeline showing year of skeletal muscle hypertrophy mechanism identified by synergist ablation (*left*) and the corresponding year that the mechanism was identified in response to human resistance exercise training (*right*). Created with Biorender.com.

In this review, we have provided a brief overview of the utility of the SA model in preclinical research. This model has been a key tool in many landmark discoveries relating to skeletal muscle hypertrophy, including, but not limited to, protein synthesis, mTOR signaling, ribosome biogenesis, and satellite cell fusion (Fig. 2). Simply put, our understanding of these processes would be incomplete and/or delayed were it not for the employment of the rodent SA model. We have also summarized the cutting-edge discoveries that are currently being elucidated with the use of SA. The SA model has not been overshadowed since its conception in 1967 but, rather, has remained at the forefront of the field.

## GRANTS

This study was supported by National Institute on Aging, National Institutes of Health, Grant/Award No. R01AG069909 (to J.J.M.).

## DISCLOSURES

No conflicts of interest, financial or otherwise, are declared by the authors.

## AUTHOR CONTRIBUTIONS

B.I.B., A.I., and J.J.M. conceived and designed research; B.I.B. and A.I. prepared figures; B.I.B. and A.I. drafted manuscript; B.I.B., A.I., and J.J.M. edited and revised manuscript; B.I.B., A.I., and J.J.M. approved final version of manuscript.
